# The bristle patterning genes *hairy* and *extramacrochaetae* regulate the development of structures required for flight in Diptera^☆^

**DOI:** 10.1016/j.ydbio.2013.12.032

**Published:** 2014-04-15

**Authors:** Marta Costa, Manuel Calleja, Claudio R. Alonso, Pat Simpson

**Affiliations:** a Department of Zoology, University of Cambridge, Downing Street, Cambridge CB2 3 EJ, UK; b Centro de Biología Molecular Severo Ochoa, C/ Nicolás Cabrera, 1, Universidad Autónoma, 28049 Madrid, Spain; c John Maynard Smith Building, School of Life Sciences University of Sussex, Brighton BN1 9QG, UK

**Keywords:** *Drosophila*, *Calliphora vicina*, *scute*, Sensory organs, Flight motor

## Abstract

The distribution of sensory bristles on the thorax of Diptera (true flies) provides a useful model for the study of the evolution of spatial patterns. Large bristles called macrochaetes are arranged into species-specific stereotypical patterns determined via spatially discrete expression of the proneural genes *achaete–scute (ac–sc)*. In *Drosophila ac-sc* expression is regulated by transcriptional activation at sites where bristle precursors develop and by repression outside of these sites. Three genes, *extramacrochaetae (emc), hairy (h)* and *stripe (sr)*, involved in repression have been documented. Here we demonstrate that in *Drosophila,* the repressor genes *emc* and *h,* like *sr,* play an essential role in the development of structures forming part of the flight apparatus. In addition we find that, in *Calliphora vicina* a species diverged from *D. melanogaster* by about 100 Myr, spatial expression of *emc, h* and *sr* is conserved at the location of development of those structures. Based on these findings we argue, first, that the role *emc, h* and *sr* in development of the flight apparatus preceded their activities for macrochaete patterning; second, that species-specific variation in activation and repression of *ac-sc* expression is evolving in parallel to establish a unique distribution of macrochaetes in each species.

## Highlights

• The distribution of sensory bristles is a useful model to study spatial patterns.
• In *Drosophila melanogaster* the genes *emc, h* and *sr* repress bristle formation.
• In *D. melanogaster emc* and *h* are essential for flight apparatus development.
• Notably, in *Calliphora vicina emc, h* and *sr* are expressed in the flight apparatus.
• We argue that *emc, h* and *sr* had an early role in flight apparatus development.

## Introduction

It is becoming clear that the evolution of developmental patterns is associated with changes in the networks of genes underlying the specification, differentiation and distribution of pattern elements. However, the specific molecular regulatory mechanisms involved and the way in which developmental networks evolve are only beginning to be explored. One mechanism for innovation is the co-option of pre-existing regulatory genes and/or networks for new roles. This has been documented in several cases, including the evolution of segmentation, heart development, butterfly wing spots, dorsal appendages of dipteran eggs and the neural crest (Keys et al., 1999; Meulemans and Bronner-Fraser, 2005; Olson, 2006; Chipman, 2009; Vreede et al., 2013). Co-option involves the rewiring of an existing gene network allowing it to affect the behavior of new cellular processes. This could occur through changes in a small number of components, such as changes in the expression domains of regulatory proteins, modification of their regulatory capacity, variation in *cis-*regulatory element composition at gene targets or changes in protein interaction domains in target proteins (Averof and Akam, 1995; Averof and Patel, 1997; Sucena and Stern, 2000; Alonso et al., 2001; Ronshaugen et al., 2002; Gompel et al., 2005; Erwin and Davidson, 2009). However identification of the molecular changes remains challenging because innovations are generally infrequent (Kopp, 2011) and their genetic analysis requires tractable experimental systems in which a morphological difference can be clearly attributed to a specific genetic alteration (Stern, 2000). The distribution of sensory bristles on the thorax of Diptera provides a useful model in which to address these questions (Simpson et al., 1999). Here we explore the possibility that an ancestral gene network has been recruited during the evolution of bristle patterns.

Many species of the sub-order Nematocera, the most ancient lineage of Diptera, display a uniform covering of randomly positioned but equally spaced bristles of similar size, a distribution thought to represent the ancestral state (McAlpine, 1981). Flies of the Cyclorrapha, a more recently derived lineage, also display uniformly spaced small bristles, microchaetes, but bear in addition large bristles, called macrochaetes, that are an evolutionary novelty of the Cyclorrapha. Macrochaetes are found in stereotypical, species-specific arrangements on the mesonotum (Simpson et al., 1999; Simpson and Marcellini, 2006). Expression of proneural genes of the *achaete–scute (ac–sc)* family (Bertrand et al., 2002) regulates development of bristle precursors and the evolution of bristle patterns correlates with evolution of the temporal and spatial expression patterns of these genes (Simpson and Marcellini, 2006). Ubiquitous proneural gene expression can account for the pattern of uniformly spaced microchaetes (Pistillo et al., 2002; Wülbeck and Simpson, 2002). In contrast, macrochaetes arise from patterned *ac-sc* expression such that discrete domains of expression prefigure the sites at which macrochaete precursors will develop (Cubas et al., 1991; Skeath and Carroll, 1991; Wülbeck and Simpson, 2000; Pistillo et al., 2002). The evolution of macrochaetes is therefore associated with the acquisition of a spatially restricted pattern of *ac-sc* expression that furthermore evolves between species.

Investigation into the genetic regulation of *ac-sc* activity in *Drosophila melanogaster* has uncovered two gene networks that are partially redundant. On the one hand the transcription factors encoded by *pannier* (*pnr*) and the genes of the *Iroquois complex* (*Iro-C*) activate transcription in the proneural clusters (Gomez-Skarmeta et al., 1996; Garcia-Garcia et al., 1999). Activation requires numerous *cis-*acting regulatory elements scattered throughout the *ac-sc* complex *(AS-C)* that appear to have evolved along with duplication events at the *AS-C* in the lineage leading to the Cyclorrapha (Gomez-Skarmeta et al., 1995; Skaer et al., 2002; Negre and Simpson, 2009). In parallel to the activators, a second set of factors antagonizes *ac-sc* function by preventing accumulation of *ac-sc* products resulting from basal promoter activity at sites outside the positions of the proneural clusters (Garrell and Modolell, 1990; Van Doren et al., 1991; Van Doren et al., 1994; Usui et al., 2008). Three antagonists have been studied, the products of the genes *stripe* (*sr*), *extramacrochaetae* (*emc*) and *hairy* (*h*). They are expressed in partially overlapping discrete spatial domains and are sufficient to correctly position bristle precursors under experimental conditions of uniform Sc expression (Rodriguez et al., 1990; Cubas and Modolell, 1992; Brand et al., 1993; Dominguez and Campuzano, 1993; Fernandes et al., 1996; Usui et al., 2008). None of these factors act via the *cis-*regulatory sequences of the *AS-C* that are the targets for Pnr and the Iro-C transcription factors (Usui et al., 2008). Thus patterning of bristles by *sr*, *emc* and *h* acts independently from patterning by activation of *ac*-*sc*.

Bristle patterns are subject to constraints imposed by structures on the thorax that are important for flight. For instance no bristles of any sort are positioned over the ridges, sutures and wing processes that are part of the flight motor (McAlpine, 1981). In addition macrochaetes, but not microchaetes, are excluded from the sites of attachment of flight muscles (Usui et al., 2004). Interestingly, the expression domains of *sr, emc* and *h* correlate with the regions from which these structures arise. So are all three genes required for the development of these structures? The flight motor of the Diptera is a highly conserved feature that was probably present in an early ancestor of this insect order long before macrochaetes appeared. If *sr*, *emc* and *h* play a role in specifying parts of the flight motor this would be likely to precede that for macrochaete patterning. It is indeed well documented that *sr* plays an important role in the development of tendons (Volk, 1999; Ghazi et al., 2003). Furthermore some of the sutures on the notum fail to form when the activity of *emc* is impaired (de Celis et al., 1995). Here we show that, in *D. melanogaster*, both *emc* and *h* are required for development of thoracic sutures, wing hinge sclerites, scutellum and scutellar lever arm. We also show that the expression of *sr, emc* and *h* in regions that give rise to the flight apparatus is conserved in *Calliphora vicina*. This is in contrast to the spatial expression of *emc* on the dorsal scutum where, like that of *ac-sc*, expression evolves in a dynamic fashion between the two species and correlates with changes in macrochaete patterns. We therefore suggest that functions of the genes related to flight are ancient and that their roles in bristle patterning might have been co-opted relatively recently in the lineage leading to the Cyclorrapha. Patterning of bristles by *emc*, *h* and *sr* would not require the evolution of any new features at the *AS-C* itself, whereas patterning through transcriptional activation is associated with gene duplication and the acquisition of numerous *cis-*regulatory elements (Skaer et al., 2002; Simpson and Marcellini, 2006; Negre and Simpson, 2009). Thus we also argue that the two mechanisms might have evolved sequentially.

## Materials and methods

### Fly rearing

*Drosophila melanogaster* flies were kept at 25 °C and fed on standard food. *Calliphora vicina* flies were kept at room temperature and fed on sucrose. Larvae were kept at room temperature and fed on minced meat.

### Gene cloning

Fragments of the genes *hairy* and *extramacrochaetae* were isolated from genomic DNA extracts from *Calliphora vicina* using degenerate PCR primers. Hairy and Emc sequences from several Dipteran species were aligned using CLUSTALW software and degenerate primer pairs were designed based on these alignments. The degenerate primers used for *hairy* were the following: Forward h_F1 5' GARAARACNGTNAARCA YYTICA 3'; h_F2 5' CARGYNGCNGA YCCIAARRT 3'; Reverse h_R1 5' CCRTTIGGNARYTTNGTNGG 3'; h_R2 5' CCANGGYCTCCANGGYTGNTCYTC 3'; h_R3 5'ACIAGISWNAGNGGYTGYTG3'.

The primers were designed for nested PCR, with h_F1 and h_R2 being the outer ones. The degenerate primers used to isolate *emc* were the following: Forward emc_F1 5'A TGAARDSNHTNACNGCIGTITG 3'; emc_F2 5' GGNGARAAYGCNGARATIMARATGTA 3'; Reverse emc_R1 5'GTRTTNGGNSWYTGICKRTC 3'; emc_R2 5' TGNCKRTCNVYNAGIGG 3'.

In this case emc_F1 and emc_R1 were the outer ones. The gene fragments obtained were cloned into pGEM-T Easy Vector (Promega) and sequenced. The identity of the fragments was verified by using BLAST with default values for algorithm parameters. In order to test for any species cross-contamination of the gene fragments obtained, specific PCR primers were designed and tested on new genomic DNA samples. Following isolation of gene fragments, the SMART^TM^ RACE cDNA Amplification Kit (Clontech) was used to obtain the complete coding region and the manufacturer's protocol was followed.

### RNA *in situ* hybridization

Digoxigenin-labelled (Roche) and/or fluorescein-labelled RNA (Roche) probes were made following standard protocols. The orthologous fragments of *hairy* and *emc* obtained by degenerate PCR primers were used as a transcription template. For *C. vicina scute* a fragment isolated by (Pistillo et al., 2002) was used. In *D. melanogaster* there are two isoforms of *sr, srA* and *srB* (Frommer et al., 1996). An orthologue of *srB* was isolated in *C. vicina* by (Richardson and Simpson, 2006). For *stripeB*, the template was a fragment of the first exon cloned from genomic DNA using the following specific primers: forward- 5' ACATGCCTGTTTAAGACCAC 3'; reverse- 5' TGTATTCAAATCTCCCTGCT 3'. For *D. melanogaster*, the 5'UTR plus the first exon of *hairy* and *emc* was used as transcription template. These fragments were isolated from genomic DNA using specific primers.

For the *in situ* hybridization, *C. vicina* larvae and white pupae wing imaginal discs were fixed according to the protocol of (Richardson and Simpson, 2006) and *in situ* hybridization was done following the protocol of (Pistillo et al., 2002), with a few modifications. Wing discs were dissected in methanol from the larval/pupal head before the start of the protocol. The digestion times with Proteinase K were changed to: L3 to 4 h after pupariation (AP) - 3 min15 s; 6 h AP to 10 h AP- 2 min30 s and discs older than 10 h AP – 1 min30 s. Samples were incubated with either anti-Digoxigenin-AP antibody (Roche) or anti-Fluorescein-AP antibodies (Roche) and color was developed with either NBT/BCIP solution (0.7 mg/ml NBT, 0.35 mg/ml BCIP) (Roche) or Fast Red Tablets (SIGMA). In the double *in situ* hybridization, samples were hybridized with both probes. The probe that was developed using Fast Red Tablets, was detected first and was incubated at twice the concentration of the other probe. After developing the first color, samples were washed 3×10 min in PBT (0.1% Tween20 in PBS) and transferred to a new tube. Samples were then rinsed in glycine buffer (50 ml: 0.375 g glycine, 500 μl 10% Tween20 in water, pH 2.0) and washed for 10 min in the same buffer. Samples were washed 3×5 min in PBX2 (PBS+ 0.2% Tween20) and blocked for 1 h in 10% normal goat serum. The protocol then followed standard procedures.

*D. melanogaster in situ* hybridization to L3 larvae wing imaginal discs was performed as described in (Negre, 2005). Expression of *hairy* was also visualized using *h1J3-Gal4/UAS-nGFP* (Bloomington: FBst0001734, (Brand and Perrimon, 1993)) wing discs, fixed in 4% paraformaldehyde, stained with phalloidine, mounted in Vectashield (VectorLaboratories) and imaged in a Leica TCS SPE laser scanning microscope.

### RNA interference protocols

The Gal4 drivers: *apMD544-Gal4* (Diaz-Benjumea and Cohen, 1993; Calleja et al., 1996; Rincon-Limas et al., 1999) and *h1J3-Gal4* (Bloomington: FBst0001734) were crossed to *UAS-dsRNA emc* (Bloomington: FBst0026738) at 25 °C, *UAS-dsRNAi h* (Bloomington: FBst0027738) at 29 °C and UAS-nGFP (Brand and Perrimon, 1993). Flies were dissected in water and mounted in Hoyer-lactic medium.

### Clonal Analysis

Females of the genotype *y w HSFlp; mwh CD2 y*^*+*^ *FRT2a/TM6B* were crossed to *emc*^*1*^ *h*^*8K2*^ *mwh FRT2A/TM6B* males (a gift from Antonio Baonza). Clones were induced by heat-shock of the progeny at 0–24, 24–48, 48–72 or 72–96 hours AEL. The genotype of the clones in males is *y w HSFlp; emc*^*1*^ *h*^*8K2*^ *mwh / emc*^*1*^ *h*^*8K2*^ *mwh*.

## Results

### stripe and scute are expressed in adjacent longitudinal stripes in *C. vicina*

**Fig. 1:**
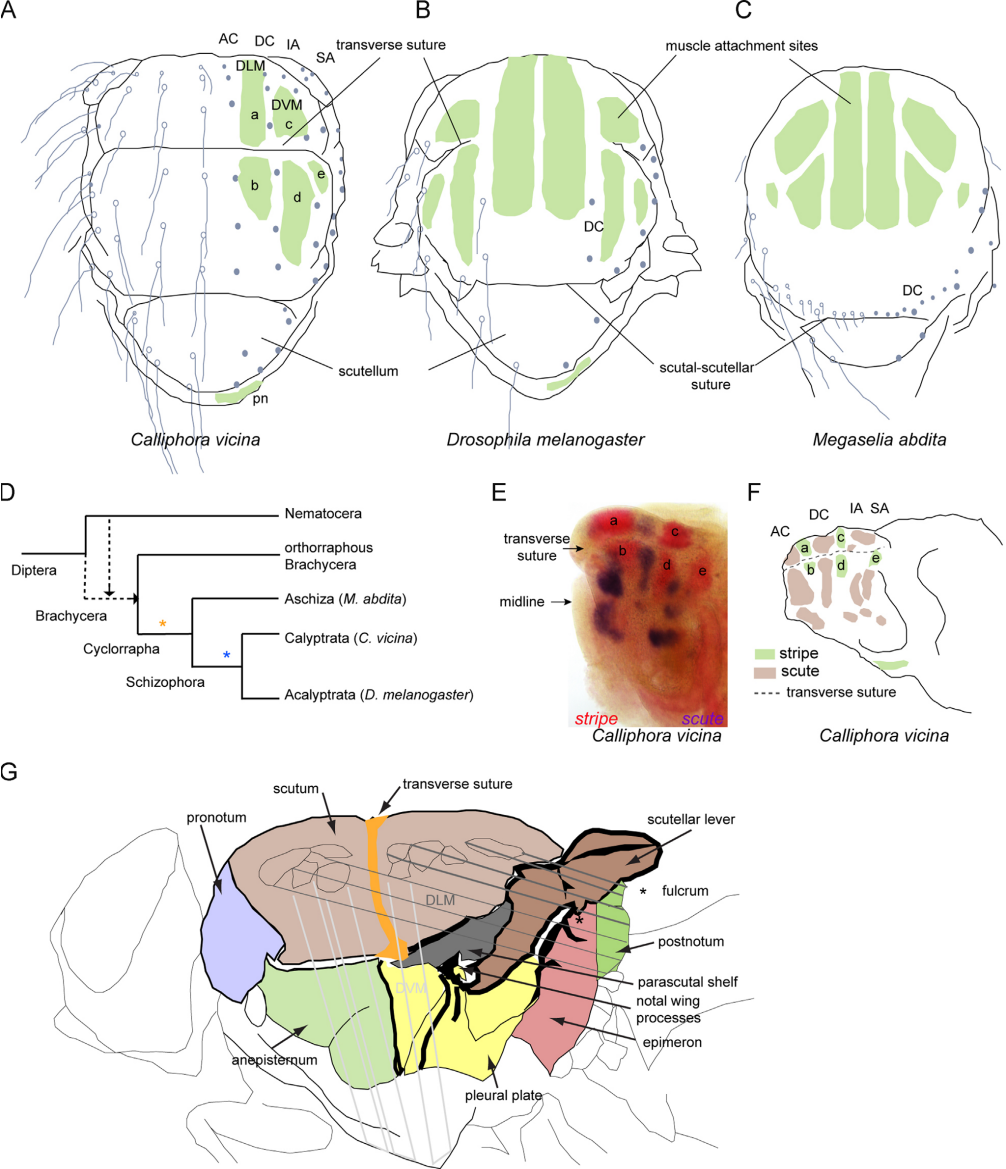
Macrochaetae and muscle attachment sites are spatially separate on the thorax of Diptera. (A), (B) and (C) The dorsal notum of Calliphora vicina, Drosophila melanogaster and Megaselia abdita with the sites of muscle attachment (green domains) and the macrochaetes (grey dots and circles). The drawings are not to scale. There is no transverse suture in M. abdita. AC, acrostichal; DC, dorsocentral; IA, intraalar; SA, supraalar; pn, postnotum; DLM, dorsolongitudinal muscles; DVM, dorsoventral muscles. The letters a, b, c, d and e refer to the domains of stripe expression to which different muscles attach, see text. (D) Simplified phylogenetic tree of the Diptera. For general details of phylogenetic groupings see (Wiegmann et al., 2011). The suborder Nematocera is probably paraphyletic and includes flies with many ancestral features. The Brachycera are monophyletic and are presumed to have arisen from some part of the Nematocera (dotted line). C. vicina and D. melanogaster belong to the Calyptrata and Acalyptrata respectively, two groups of Schizophora separated by about 100 Myr of divergence (blue star). Macrochaetes probably arose in the lineage leading to the Cyclorrapha (yellow star). (E) Double *in situ* hybridization showing the expression domains of scute (violet) and stripe (red) in the presumptive hemithorax of C. vicina at 2 h APF (the thorax is derived from two imaginal discs each of which comprises one wing and a hemithorax). (F) Drawing of the thoracic disc indicating the correspondence of the expression domains of scute (brown) to the rows of bristles and of stripe (green) to the sites of muscle attachment. (G) A lateral view and sagittal sections of a generalized thorax of the Calyptrata (modified from (Miyan and Ewing, 1985)). It is composed of the pronotum, the dorsal scutum and scutellum, which is on top of the postnotum, together with the anepisternum, pleural plate and epimeron on the lateral sides, which in turn are joined ventrally to the sternum, a product of the leg discs. The scutellar lever is composed of the scutellum and the anterior ventral arm that terminates in the posterior notal wing process. The fulcrum (* in A) for rotation of the scutellar lever is a set of ridges bounding the epimeron. The section on the left is at the level of the transverse suture, a thickened ridge that terminates in the anterior notal wing process at the level of the wing articulation. The dorsolongitudinal muscles (DLM), composed of six large fibers, have their anterior ends attached to the scutum and their posterior ends attached to the post-notum and epimeron. The dorsoventral muscles (DVM) have their anterior ends attached to the lateral scutum and their posterior ends to the sternum. The red dot indicates the pleural wing process.

A side view of the thorax of a typical cyclorraphan fly is shown in Fig. 1G. The cuticular plates, sutures, wing processes and positions of underlying flight muscles are indicated. These vary little between species. A dorsal view of the thorax showing the positions of the sites of muscle attachment and the macrochaetes of *C. vicina* and *D. melanogaster* are shown in Fig. 1A,B. *C. vicina* is a species of calyptrate Schizophora diverged from *D. melanogaster* by about 100 Myr (Fig. 1D) (Wiegmann et al., 2011). It displays a pattern of four rows of macrochaetes on the scutum (the acrostichal (AC), dorsocentral (DC), intraalar (IA) and supraalar (SA)) and has become a useful species to compare with *D. melanogaster*, which is lacking AC and IA bristles and bears a reduced number of DC and SA bristles (Fig. 1B) (Simpson et al., 1999; Pistillo et al., 2002). The macrochaetes are located outside the sites of muscle attachment, a feature found throughout the Cyclorrapha (Usui et al., 2004).

The pattern of indirect flight muscles and their sites of attachment are conserved throughout the Diptera (Tiegs, 1955). The muscles attach via tendons whose precursor cells develop in the wing/thoracic disc from the same epithelium as the bristle precursors (Huang et al., 1991; Fernandes et al., 1996; Volk, 1999). The development of tendon precursor cells is preceded by expression of *sr,* whose product, a transcription factor, activates genes required for tendon development (Volk and VijayRaghavan, 1994; Ghazi et al., 2003). *stripe* is expressed in a conserved pattern of longitudinal domains in the presumptive notum, that prefigures the sites of muscle attachment in *D. melanogaster* and *C. vicina* (Fernandes et al., 1996; Usui et al., 2004; Richardson and Simpson, 2006). Expression of both *sr* and *sc* in *D. melanogaster* starts at mid third larval instar, whereas in *C. vicina* it is delayed until the onset of pupariation. In *D. melanogaster,* at the time of macrochaete precursor development, expression of *ac-sc* and *sr* is mutually exclusive (Usui et al., 2004). Sequences corresponding to *sc* and *sr* from *C. vicina* were already available (Richardson and Simpson, 2006); here we have performed double *in situ* hybridization in order to determine the relative domains of expression of *sr* and *sc* in *C. vicina*.

Expression of *sr* was found to be similar to the pattern previously described for both *D. melanogaster* and *C. vicina* (Fernandes et al., 1996; Usui et al., 2004; Richardson and Simpson, 2006) (Fig. 1E). Two expression domains, (a) and (b), correspond to the region where the dorsal longitudinal muscles (DLM) attach at their anterior ends; they are separated by the transverse suture. Expression in the prospective postnotum marks the posterior attachment sites of the DLMs. Expression domains (c) and (d) pre- figure the dorsal attachment sites for the dorsoventral muscles (DVM). The weaker lateral domain (e) marks the anterior attachment site for the tergal depressor of the trochanter of the second leg (the jump muscle). Double staining with *sc* revealed that the domains of expression of *sr* and *sc* in *C. vicina* are complementary, but not juxtaposed (Fig. 1E,F). The band of *sc* expression corresponding to the AC row of bristles is dorsal to the *sr* (a-b) domains, that corresponding to the DC row is in between domains (a-b) and (c-d), and finally the IA and SA rows are in between (c-d) and the attachment site of the tergo- trochanteral muscle.

### Isolation of sequences corresponding to extramacrochaetae and *hair*y from Calliphora vicina

Sequences corresponding to *emc* and *h* were isolated from *C. vicina* by degenerate primer PCR and RACE (Suppl. Fig. 1). The gene *h* encodes a transcriptional repressor of *ac-sc*, belonging to the conserved basic helix-loop-helix (bHLH) superfamily of transcription factors (Carroll and Whyte, 1989; Rushlow et al., 1989; Ohsako et al., 1994; Van Doren et al., 1994). The *h* protein of *C. vicina* displays more than 60% identity with that of *D. melanogaster*. The Hairy/Enhancer of split subfamily contain other discrete domains (orange domain, HC domain, and a conserved WRPW motif at the C-terminal end of the protein) and are distinguishable by a conserved proline residue (Paroush et al., 1994; Dawson et al., 1995; Fisher and Caudy, 1998; Davis and Turner, 2001). These features are well conserved, in addition to two other stretches of amino acids. The product of *emc* is an HLH protein devoid of a basic domain, which sequesters Ac-Sc in the cytoplasm (Ellis et al., 1990; Garrell and Modolell, 1990; Van Doren et al., 1991; Martinez et al., 1993). The *C. vicina* homolog of Emc is less well conserved than that of H: 56% identity.

### *Hairy* is expressed in a conserved domain that covers the scutellar suture and the anterior ventral arm of the scutellar lever

**Fig. 2:**
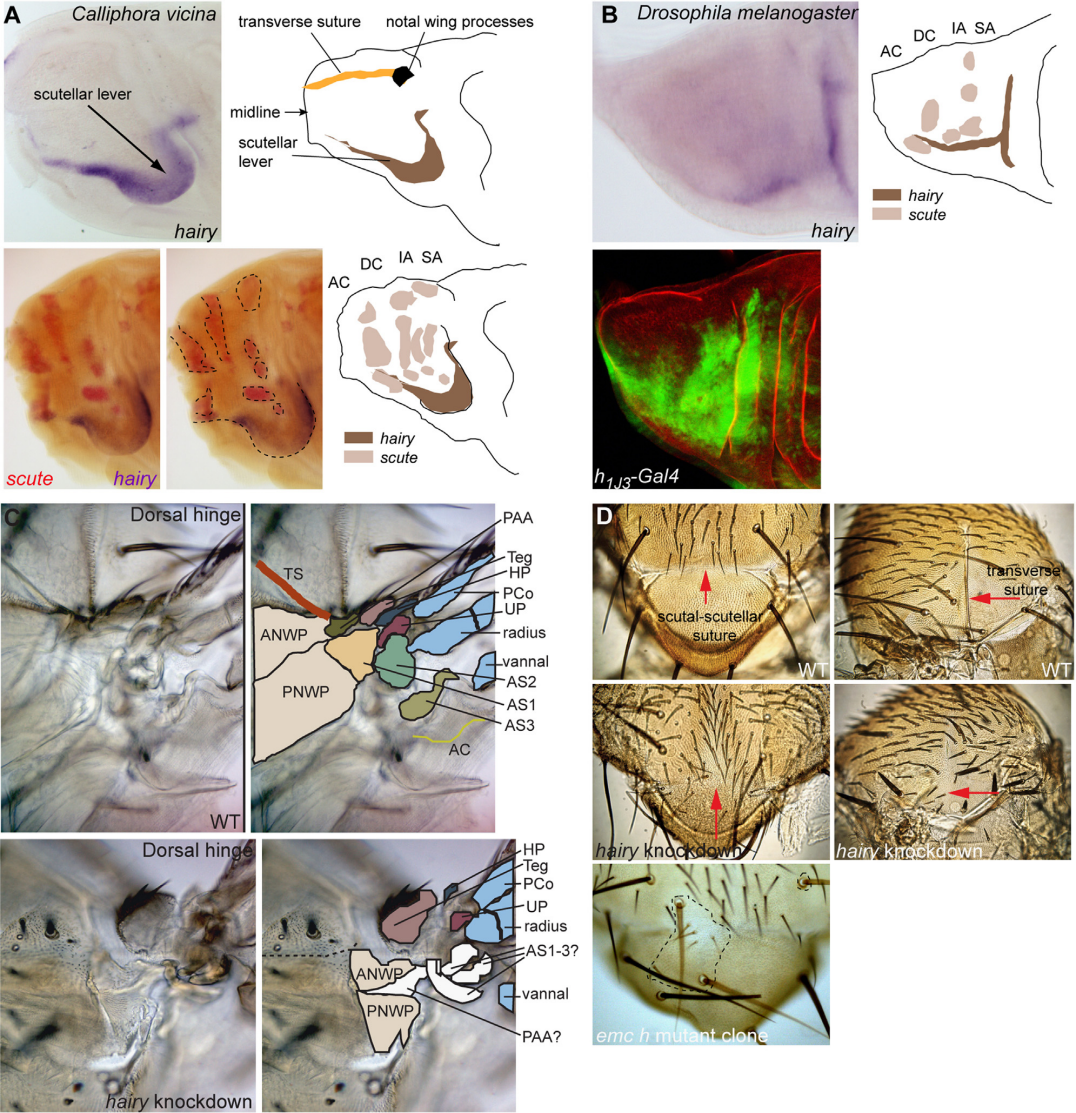
hairy is expressed at the sites of the scutellar lever and wing hinge and is required for the normal development of these structures. (A) Expression of hairy (h) in the wing/thoracic disc of Calliphora vicina visualized by *in situ* hybridization at 3 h APF. From a fate map constructed by Sprey (Sprey and Oldenhave, 1974), it can be seen that expression of h corresponds to the anterior border of the scutellar lever, which forms a prominent bulge in the disc at the stage shown. Double *in situ* hybridization for scute and h in Calliphora vicina at 2 h APF and diagram showing an interpretation. There is very little overlap between the expression domains of the two genes. (B) *in situ* hybridization for h in Drosophila melanogaster at third larval instar and expression revealed in h1J3-Gal4/UAS-nGFP discs. The diagram shows an interpretation of position of the h expression domain relative to the domains of scute expression known from previous studies. (C) Dorsal wing hinge region of wild type (WT) and apMD544- Gal4/UAS-dsRNA h flies. The images have been duplicated and the colored regions on the right indicate specific structures. It can be seen that the mutant wings are poorly formed and many of the sclerites cannot be identified, some appear to be absent and others are deformed. (D) Dorsal (left panels) and lateral views (right panels) of the nota of wild type (WT) and apMD544-Gal4/UAS-dsRNA h flies (hairy knockdown). Below a fly bearing a clone mutant for emc^1^ h^8Ka^. Red arrows indicate the scutal-scutellar suture and the transverse suture. The sutures are missing or only partially formed in the knockdown or mutant clone. PAA: pre-alar apophysis; Teg: tegula; HP: humeral plate; PCo: proximal costa; radius and vannal: wing veins; AS1, AS2, AS3: axillary sclerites; AC: axillary cord; AP: axillary pouch; PS: pleural sclerite; PWP: pleural wing process; YC: yellow club; PVR: proximal ventral radius. For a complete description of the terminology of the wing base structures see (Bryant, 1978).

At the time of macrochaete precursor development in *D. melanogaster, in situ* hybridization reveals that the gene *h* is strongly expressed in a stripe that extends transversely just above the presumptive scutellum, and then curves and extends anteriorly up to the position of the future notal wing processes (Fig. 2B) (Bryant, 1975; Carroll and Whyte, 1989; Usui et al., 2008). It is also expressed in parts of the wing hinge. Expression levels of mRNA or antigen (Carroll and Whyte, 1989) are low, so expression was also examined in *h1J3-Gal4/UAS-nGFP* discs. This revealed an expanded area of expression over the dorsal notum as well patches of expression in regions of both the dorsal and ventral wing hinge (Fig. 2B). Expression of *h* in *C. vicina* starts at the last larval instar in a transverse stripe posterior to the stripes of *sc* expression on the scutum, and is the same at all stages (Fig. 2A). It is restricted to a distinct stripe that outlines a fold of the disc that becomes a prominent bulge by 10 h AP. The fold appears to define the site where the scutellum and scutellar lever arm develop, according to the fate map constructed by Sprey and Oldenhave (1974). Expression of *h* thus appears to define the anterior boundary of the scutellar lever (see Discussion). This is a site where bristles are never located in Diptera (McAlpine, 1981), consistent with the lack of overlap between *h* and *sc* expression (Fig. 2A).

### *hairy* is required for development of the sutures, scutellum, scutellar lever and wing hinge in Drosophila

A phenotype of ectopic microchaetes on the notum and wing has been described for viable mutant alleles of *h* in *D. melanogaster* (Moscoso del Prado and Garcia-Bellido, 1984; Ingham et al., 1985; Rushlow et al., 1989; Usui et al., 2008). However *h* is strongly expressed over the scutellum and scutellar lever arm, an area of expression that is conserved in *C. vicina.* So does *h* play a role in the development of these structures? One possibility is to examine clones of mutant alleles that are otherwise lethal. The scutellar lever arm terminates in the wing processes that are part of the wing hinge. Notably the cuticle of these structures is mostly devoid of hairs and bristles such that markers for clonal analysis are not available. Clones doubly mutant for *emc*^*1*^ *h*^*8Ka*^ and marked with *yellow* were examined. The scutal-scutellar and transverse sutures were found to be missing (Fig. 2D). However, although it could be seen that clones overlapping the hinge region resulted in abnormal hinge structures, these proved too difficult to interpret. Therefore loss of function of *h* has been studied using RNA interference. Two *UAS-RNAi h* lines and various *Gal-4* drivers were tested and the resulting phenotypes were variable in strength between lines and from one animal to another but were consistent. The strongest phenotype was observed with *apMD544- Gal4* which drives expression over the entire dorsal notum and wing (Diaz-Benjumea and Cohen, 1993; Rincon-Limas et al., 1999) and *UAS-dsRNAi h* BL27738 at 29 °C. Ectopic bristles were seen on the wing and notum including the scutellum, as previously described for *h* loss of function. The scutellum is reduced in size and somewhat flattened and the scutal-scutellar suture is missing (Fig. 2D). Bristles are present at the site where the suture normally resides. The transverse suture is incomplete (Fig. 2D). It is present medially but fails to form over the lateral notum where it normally meets the pleura and extends into the anterior notal wing process. Indeed the lateral notum where the anterior and posterior wing processes are found is reduced in size. The anterior notal wing process is present but is deformed and the posterior notal wing process cannot be distinguished, so that the articulation between the two appears to be non functional (Fig. 2C). This may explain the fact that the wings are held up, and probably means that the wing beat is compromised. The animals are unable to fly. The tegula is present but the pre-alar apophysis appears to be absent. The three axillary sclerites are present but are mis-shapen and difficult to discern. Components of the ventral wing hinge region are all present and this region is only slightly distorted (not shown).

### extramacrochaetae is expressed in five transverse stripes on the dorsal notum of *C. vicina*

**Fig. 3:**
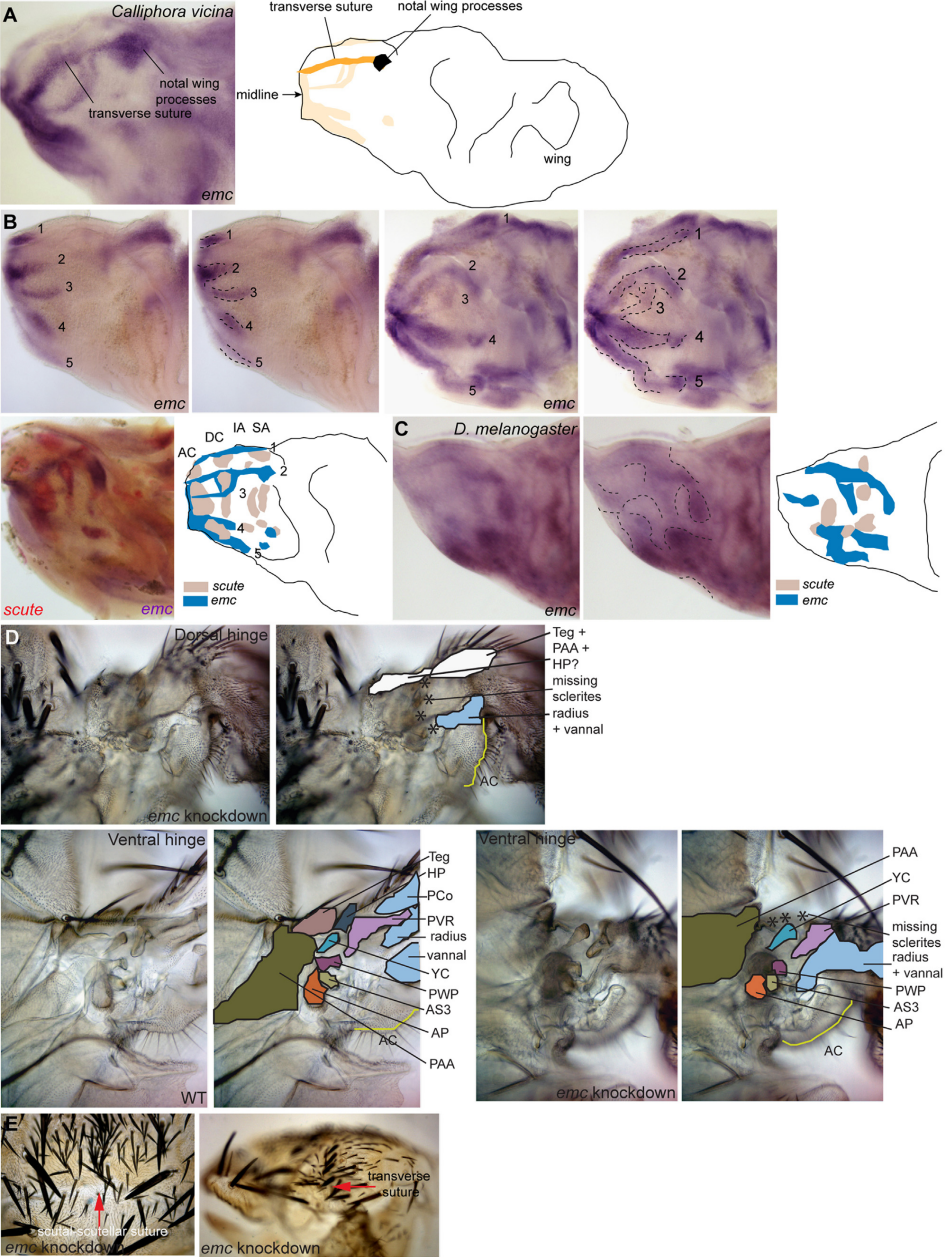
*extramacrochaetae* is expressed at the sites of the sutures and wing hinge and is required for the normal development of these structures. (A) Expression of *emc* in the wing/thoracic disc of *Calliphora vicina* visualized by *in situ* hybridization at the white prepupal stage defines the transverse suture and the notal wing processes. (B) *In situ* hybridization for *emc* at third larval instar and at 6 h APF, and double *in situ* hybridization for *scute* and *emc* at 4 h APF in *Calliphora vicina*. There are few areas of overlap. (C) *In situ* hybridization for *emc* at third larval instar in *Drosophila melanogaster* and diagram showing an interpretation together with the known domains of *scute* expression. This is based on single *in situs* but also on double labeling of *emc* and a bristle precursor marker performed by (Cubas and Modolell, 1992). (D) Dorsal and ventral wing hinge region of the same specimen of an *apMD544-Gal4*/*UAS-dsRNA emc* fly. The images have been duplicated and the colored regions on the right indicate specific structures. For the WT see Fig. 2. It can be seen that the mutant wings are poorly formed and many of the sclerites cannot be identified, some appear to be absent and others are deformed. (E) Dorsal and lateral views of nota of *apMD544-Gal4*/*UAS-dsRNA emc* flies. For the WT see Fig. 2. Red arrows indicate the positions of the scutal-scutellar and transverse sutures that are missing. For labels see Fig. 2.

In *D. melanogaster, emc* is expressed ubiquitously but the levels vary in a complex, dynamic pattern (Cubas and Modolell, 1992) (Fig. 3C). Expression partly overlaps with that of *h* in the region of the scutellum, scutellar lever and wing hinge. Although a discrete, evolving pattern of strong expression is clearly visible in *C. vicina, emc* seems to be expressed at low levels throughout the disc. For this reason, the *in situ* reaction development time had to be carefully monitored. For double staining the *emc* reaction could not be developed as strongly. At some locations expression of *emc* appears to be conserved with that of *D. melanogaster* and furthermore corresponds to the sites of development of specific structures. Expression is strong over the presumptive transverse suture, in a transverse band, in both species and only differs in that the suture extends across the entire scutum in *C. vicina* but is partial in *D. melanogaster* (Fig. 3A). At the lateral end of this band two domains become visible at the positions where the posterior and anterior notal wing processes develop (Fig. 3A) (Sprey and Oldenhave, 1974; Miyan and Ewing, 1985). There is also a clear domain lateral to these processes corresponding to the location of the tegula (not shown). There are furthermore many small discrete domains of expression of *emc* in the presumptive wing hinge where the wing processes and other sclerites form.

Over the dorsal notum, expression of *emc* differs significantly between the two species. From white prepupae to 10 h APF *emc* is expressed in five transverse bands over the dorsal notum of *C. vicina* (Fig. 3B). These appear gradually from the medial side from stage L3. The first is at the anterior edge of the prescutum where the scutum will later fuse with the pronotum. The second band corresponds to the transverse suture. The third band is midway down the scutum. Band four is at the level of the scutal-scutellar suture. Band five is at the posterior edge of the future scutellum where the scutellar lever is joined to the postnotum. In addition to the five transverse bands some smaller longitudinal bands and domains become visible from pupariation. The domains of *ac-sc* and *emc* expression are largely complementary in *D. melanogaster* (Cubas and Modolell, 1992) (Fig. 3C). Similarly, double staining with *emc* and *sc* in *C. vicina* reveals a pattern of mostly complementary gene expression with only a few sites of overlap (Fig. 3B). Most notably the stripes of *sc* expression are perpendicular to those of *emc,* forming a grid-like pattern.

Double *in situ* hybridization with *emc* and *sr* reveals complementary domains of expression (Suppl. Fig. 2).

### extramacrochaetae is required for the formation of sutures and wing hinge processes in Drosophila

Viable hypomorphs of *emc* display weak phenotypes of ectopic bristles (Moscoso del Prado and Garcia-Bellido, 1984; Usui et al., 2008). A total loss of function is however cell lethal (Garcia-Alonso and Garcia-Bellido, 1988). This, together with the difficulty of marking clones in the hinge region prompted us to examine loss of function of *emc* using RNA interference. Two *UAS-RNAi emc* lines and the *ap-Gal-4* driver were employed. The resulting phenotypes vary in strength from cross to cross but are consistent in nature. Phenotypes previously described for *emc* loss of function were observed. There are numerous ectopic bristles and both the transverse suture and the scutal-scutellar suture fail to form (Fig. 3E). The wing hinge is not properly formed. The dorsal hinge is highly abnormal: the anterior and posterior notal wing processes and the axillary sclerites cannot be identified within a distorted, twisted cuticle, the dorsal radius and vannal veins are fused proximally and the prealar apophysis, tegula and humeral plate also merge into a single, poorly defined structure (Fig. 3D). The ventral hinge is less affected: some of the sclerites are recognizable although they are misshapen (Fig. 3D). The animals are unable to fly.

## Discussion

### Extramacrochaetae, hairy and stripe play a role in development of parts of the flight motor

Wing movement in flies is caused by a deformation of the thorax brought about by contraction of the indirect flight muscles, which are attached to the thoracic cuticle (Miyan and Ewing, 1985) (Fig. 1G). The scutellar lever is a structure consisting of the scutellum and anterior ventral arm (Fig. 1G). The anterior edge of the scutellar lever is thickened to form the posterior notal wing process, which articulates with another sclerotized region, the anterior notal wing process, via a series of axillary sclerites. Contraction of the dorsal longitudinal muscles (DLM) causes a rotation of the scutellar lever, raising the posterior notal wing process, which rotates about its articulation with the anterior notal wing process until it stops against a sclerotized region of the parascutal shelf and the pleural wing process. This causes the roof of the scutum to arch upwards and the wings to make a downward stroke. Contraction of the dorsoventral muscles (DVM), which run perpendicular to the DLMs, reverses the deformation of the scutum producing a stretching of the DLMs and the causing the wings to make an upward stroke (Miyan and Ewing, 1985). To accommodate the changes in shape brought about by contraction of the muscles, the thorax is essentially a cage with walls that are stiff in some places and flexible in others (Fig. 1G). There are a number of strengthening sclerotized cuticular ridges and plates as well as flexible sutures. The transverse “suture” is a structural ridge visible externally, which gives greater strength to the scutum and which terminates in the anterior notal wing process (Fig. 1G). In contrast the scutal-scutellar “suture” is a flexible membrane that accommodates the up and down movement of the scutellum (Fig. 1G).

Our work and that of others demonstrate, that, in *D. melanogaster*, *h, sr* and *emc* are all required for the development of the flight apparatus. The sites of attachment of the indirect flight muscles in *D. melanogaster* are specified by expression of *sr,* a gene whose activity is essential for tendon development (Fernandes et al., 1996; Volk, 1999). In the absence of tendons the muscles do not attach to the cuticle and therefore flight is impossible. When activity of *h* is impaired, development of the scutellum, the scutellar lever arm and the wing processes is abnormal, the sutures are partially absent and the wings are maintained in a ‘held up’ position. When *emc* activity is reduced, the cuticular ridges and sutures are absent, many of the sclerites in the wing hinge are missing or distorted and the wings are unable to articulate. In both cases the animals cannot fly. We conclude that, in *D. melanogaster, h, emc* and *sr* are all required for the development of structures related to flight.

An obvious question is whether the function of *emc, h* and *sr* is conserved in other species? The flight motor with its attendant pattern of muscles and cuticular structures is largely unchanged throughout the Diptera (Tiegs, 1955; McAlpine, 1981). In fact the overall structure of the scutum, which is the most obvious component of the dorsal thorax, is an outstanding apomorphic character of the order Diptera (McAlpine, 1981). The scutellum is always a clearly defined lobe at the posterior margin of the scutum and the axillary region of the wing hinge is largely conserved (McAlpine, 1981). Similarly little variation in the patterning of indirect flight muscles and the positioning of tendons is seen throughout the order (Tiegs, 1955; Levine and Hughes, 1973; Usui et al., 2004). Only the transverse suture displays some variability. It is often weakly formed in the Nematocera and absent in some basal cyclorraphans such as *Megaselia abdita* (Fig. 1C,D). In most Calyptratae and some Acalyptratae the suture is more strongly transverse; it extends across the entire width of the scutum in *C. vicina* but is only partial in *D. melanogaster*.

To address the question of conservation of the underlying genetic networks we have examined the expression patterns of *emc, h* and *sr* in *C. vicina,* a species diverged from *D. melanogaster* by about 100 Myr. Expression of *sr* is conserved between *D. melanogaster* and *C. vicina* (Fernandes et al., 1996; Usui et al., 2004; Richardson and Simpson, 2006). Expression of *emc* at the sites of development of cuticular ridges and sutures is very obvious in *C. vicina* where five transverse bands of expression are seen on the presumptive dorsal notum. The first is at the point where the prescutum joins the pronotum where there is thought to be a line of weakness in the cuticle to accommodate the upward movement of the scutum at the wing downbeat. Other bands correspond to the transverse suture, the scutal-scutellar suture and the posterior edge of the scutellum where the scutellar lever is joined by a flexible cuticle and membrane to the postnotum. Expression of *h* at the site of the presumptive scutellar lever is also conserved. The conservation of gene expression in *C. vicina* makes it likely that the roles of *emc, h* and *sr* is conserved, although functional studies would be required for a definitive answer.

### The role of extramacrochaetae, hairy and stripe in the development of the flight motor might predate their function for bristle patterning

**Fig. 4:**
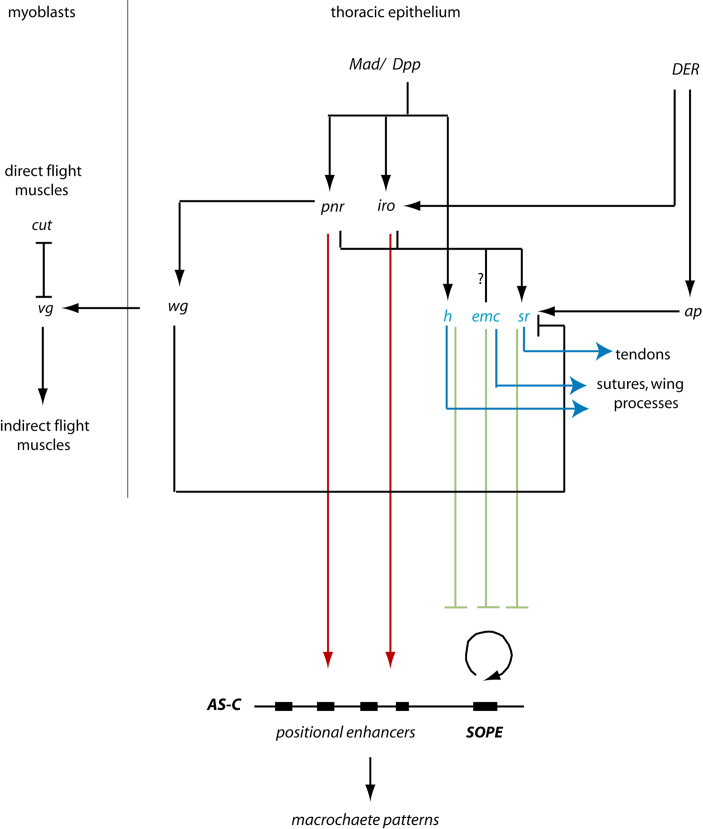
*Gene regulatory network patterning the thorax in D. melanogaster.* The thorax is patterned by a gradient of Dpp/TGF-ß. Downstream of Dpp, two selector genes, *pnr* and the *IRO-C* genes, pattern the medial and lateral halves of the notum respectively (Calleja et al., 2000; Cavodeassi et al., 2000; Mann and Morata, 2000). Their activity is probably conserved in the Diptera (Wülbeck and Simpson, 2002; Richardson and Simpson, 2006). A The three genes *h, emc* and *sr* (shown in blue) play a role in patterning the tendons, sutures and wing processes. Spatial expression of *h* coincides with that of phospho-Mad and is reduced in *dpp* mutants (unpublished observations), so *h* is likely to be directly activated by the Dpp signaling pathway, as it is in the leg (Kwon et al., 2004). Spatial expression of *emc* appears also to be dependent on *pnr* and the *IRO-C* genes (Costa, 2011). Activation of *stripe* is dependent on *pnr* and *IRO-C* (as well as on *apterous,* a dorsal selector gene of the wing/thoracic disc) (Ghazi et al., 2003; Ikmi et al., 2008). Refinement of *sr* expression to distinct domains is dependent on repression by Wingless signaling (Piepenburg et al., 2000; Ghazi et al., 2003). Expression of *wg* is dependent on Pnr (Couso et al., 1993; Sato and Saigo, 2000; Tomoyasu et al., 2000). Wingless signaling has another essential function: it subdivides the flight muscles, which develop from myoblasts underlying the thoracic disc, into two groups for the direct and indirect flight muscles (Sudarsan et al., 2001). Expression of *wg, h, emc* and *sr* is conserved between *C. vicina* and *D. melanogaster* suggesting they are part of an ancient regulatory network for development of the flight apparatus in Diptera (Richardson and Simpson, 2006). Products of *emc, h* and *sr* also play a role in patterning the bristles, shown in green. They negatively regulate *ac-sc* expression via an autoregulatory element, the *SOPE* (Jarman et al., 1993; Martinez et al., 1993; Van Doren et al., 1994; Culi and Modolell, 1998; Usui et al., 2008; Ayyar et al., 2010). We propose that they have been co-opted for *ac-sc* regulation in the lineage leading to the Cyclorrapha. In parallel the products of *pnr* and *IRO-C* directly activate *ac-sc* via discrete *cis-*regulatory sequences (positional enhancers) that are also thought to have originated in the lineage leading to the Cyclorrapha (shown in red) (Gomez-Skarmeta et al., 1995, 1996; Garcia-Garcia et al., 1999).

If the functions of *emc, h* and *sr* in the specification of the flight motor are evolutionarily ancient, did this ancestral function predate a role in patterning the bristles? Throughout the Diptera bristles are absent from the sutures, the flight lever and the wing processes (McAlpine, 1981). Thus *emc* and *h* might have had a functional link with the *ac-sc* genes to prevent bristles at these locations early in dipteran evolution (Fig. 4). Indeed an ancient transcriptional regulatory link between *hairy* and *ac-sc* has been documented (Rebeiz et al., 2005; Ayyar et al., 2010). A function of *sr* to prevent the formation of macrochaete precursors is, however, likely to be more recent. A role for *sr* in the development of tendons was probably inherited from an early dipteran ancestor, but bristles in Nematocera do form over the muscle attachment sites, as do the microchaetes of cyclorraphous flies in spite of the expression of *sr* (McAlpine, 1981; Usui et al., 2004).

Macrochaetes are an evolutionary novelty associated with the Cyclorrapha, that, unlike microchaetes and the bristles found in basal groups, are invariably present in specific arrangements on the dorsal scutum (Simpson et al., 1999). Unlike other structures on the notum, macrochaete patterns evolve between species. Furthermore, our results indicate, that, on the dorsal scutum, the expression domains of *h* and *emc* evolve between species and correlate negatively with the positions of the bristles. This is in contrast to their conserved expression domains at sites where the flight apparatus develops. In *D. melanogaster emc, h* and *sr* are all required for the precise positioning of macrochaetes (Cubas and Modolell, 1992; Huang et al., 1995; Usui et al., 2008). Therefore one possibility is that the three genes were already expressed on the notum for patterning the flight apparatus (and in the case of *emc* and *h* for preventing bristle development there) and have been co-opted for macrochaete patterning in the lineage leading to the Cyclorrapha (Fig. 4). This is likely to have required changes in the spatio-temporal expression of *emc* and *h* as well as a novel linkage between *sr* and targets of the *ac-sc* genes. Co-option of gene regulatory networks for evolution of novel morphologies is an emerging theme in pattern evolution. Examples include co-option of networks specifying body axes for regulating segmentation (Chipman, 2009), co-option of new regulatory inputs into the ancestral cardiac transcription factors during evolution of heart complexity (Olson, 2006), the co-option of an ancestral wing patterning circuit in the evolution of butterfly wing spots (Keys et al., 1999), co-option of pre-existing signals in the evolution of dorsal appendages on the eggshell of Diptera (Vreede et al., 2013) and the recruitment of new pathways during evolution of neural crest development (Meulemans and Bronner-Fraser, 2005).

### Evolution of the regulation of *achaete*–*scute* activity

In parallel to patterning by *h, emc* and *sr*, expression of *ac–sc* in spatially defined domains in *D. melanogaster* is also regulated by direct transcriptional activation. Products of the *pnr* and the *IRO-C* genes activate transcription through a number of independently acting *cis-*regulatory sequences at the *AS-C* (Gomez-Skarmeta et al., 1995, 1996; Garcia-Garcia et al., 1999). The two patterning mechanisms are largely independent raising the possibility that they could have evolved separately. If so could one of them predate the other? The molecular means by which the products of *emc, h* and *sr* antagonize bristle development would not, in theory, have required the evolution of any new features at the *AS-C* itself. Alone of the three, Hairy is a transcriptional repressor but it acts via an auto-regulatory element, the Sensory Organ Precursor Element (SOPE), that allows accumulation of high levels of Ac-Sc in sensory organ precursors (Jarman et al., 1993; Martinez et al., 1993; Van Doren et al., 1994; Culi and Modolell, 1998). This is the only *cis-*regulatory element at the *AS-C* that has been shown to predate the Diptera (Ayyar et al., 2010). Emc and Sr prevent bristle formation by interfering with the accumulation of *ac-sc* proteins required for formation of bristle precursors (Ellis et al., 1990; Garrell and Modolell, 1990; Usui et al., 2004, 2008). As *emc, h* and *sr* act at a step downstream of the initial transcription of *ac-sc,* they could have patterned the bristles in ancestral species in which *ac-sc* was expressed uniformly. Indeed they are able to correctly pattern the macrochaetes in *D. melanogaster* under experimental conditions of ubiquitous *sc* expression (Rodriguez et al., 1990; Usui et al., 2008). In contrast, the second mechanism of bristle patterning via spatially restricted transcriptional activation of *ac-sc* is likely to have accompanied evolution of the *AS-C* itself. The *cis-*regulatory elements are thought to be of relatively recent origin and to have been acquired along with gene duplication events at the *AS-C* that occurred during the evolution of the lineage leading to the Cyclorrapha (Skaer et al., 2002; Simpson and Marcellini, 2006; Negre and Simpson, 2009). Antagonism of Ac-Sc activity by the products of *emc, h* and *sr* could therefore predate patterning of *ac-sc* expression through transcriptional activation.

The species-specific patterns of macrochaetes are thought to be variations of a bauplan of four longitudinal (anterior-posterior) rows (Simpson et al., 1999). Four longitudinal bands of *sc* expression interspersed with bands of expression of *sr* would have been at the origin of this pattern (Pistillo et al., 2002; Usui et al., 2004). Expression of *emc* on the dorsal scutum of *C. vicina* is roughly in five transverse bands, perpendicular to the bands of *sc* expression, so *emc* might be responsible for the positioning of bristle precursors along the anterior-posterior axis within the rows. Thus a grid-like pattern of intersecting stripes of *sc* and *emc* gene products could have underpinned the origin of an ancestral arrangement of macrochaetes. Patterns in many species of Acalyptrata are a result of a complete or partial loss of one or more rows. Evolution of *cis-*regulatory sequences at the AS-C at least partially underlies these different bristle patterns (Garcia-Garcia et al., 1999; Marcellini and Simpson, 2006). However expression of *emc* on the dorsal scutum has diverged quite significantly between *D. melanogaster* and *C. vicina* suggesting that *emc* also plays a role in the evolution of the patterns. It is not known how expression of *emc* is regulated spatially, but preliminary results with *D. melanogaster* indicate that its expression is altered in *pnr* and *IRO-C* mutants (Costa, 2011). If so, this could mean that *emc* is regulated by precisely the same transcription factors that activate *sc*. Expression of pnr and IRO-C genes is conserved between *C. vicina* and *D. melanogaster* suggesting they are part of an ancient regulatory network patterning the dipteran thorax (Mann and Morata, 2000; Richardson and Simpson, 2006) (Fig. 4). Therefore perhaps both *emc* and *sc* are evolving in response to the same *trans-*regulatory prepattern: *sc* to be present at the sites of the future bristles and *emc* to be present in a complementary pattern where no bristles develop.

## Appendix A Supplementary materials

Supplementary Material **Supplementary Figure 1** Sequence of *hairy* and *extramacrochaetae* from *Calliphora vicina*. (A) Protein alignment of *hairy*. The protein domains are indicated: bHLH (blue), Orange (Orange), HC (green) and WRPW (grey). (B) Protein alignment of *extramacrochaetae*. The HLH protein domain is indicated in blue.

Supplementary Material **Supplementary Figure 2** Expression of *extramacrochaetae* and *stripeB* in *Calliphora vicina,* visualized by double *in situ* hybridization. (A) Double *in situ* hybridization showing the expression domains for *extramacrochaetae* (blue) and *stripe* (red) in the presumptive thorax at 2 h APF. (B) Drawing of the thoracic disc indicating the expression domains of *scute* (brown), *extramacrochaetae* (blue) and of *stripe* (green). *extramacrochaetae* and *stripe* are mostly complementary. AC, acrostichal; DC, dorsocentral; IA, intraalar; SA, supraalar; pn, postnotum
