# Supplementary material for: The bristle patterning genes hairy and extramacrochaetae regulate the development of structures required for flight in Diptera
Source: Dev Biol. 2014 Apr 15;388(2):205–15. doi: 10.1016/j.ydbio.2013.12.032 (PMC3988846; doi:10.1016/j.ydbio.2013.12.032)
Supplement: Supplementary file 1 — Supplementary Material Supplementary Figure 1 Sequence of hairy and extramacrochaetae from Calliphora vicina. (A) Protein alignment of hairy. The protein domains are indicated: bHLH (blue), Orange (Orange), HC (green) and WRPW (grey). (B) Protein alignment of extramacrochaetae. The HLH protein domain is indicated in blue. [file mmc1.pdf]

SUPP. FIGURE 1

a

```
Dmel_h          MVTGVTAANMTNVLGTAVVPAQLKETPLKSDRRSNKPIMEKRRRARINNCLNELKTLILD 60
Cvic_h          MVTGVCGPTMTANVIGAPISHPPKETPAKSDRRSNKPIMEKRRRARINNCLNELKTLILD 60
                ***** ...**  :  * :.   **** ********************************

Dmel_h          ATKKDPARHSKLEKADILEKTVKHLQELQRQQAAMQQAADPKIVNKFKAGFADCVNEVSR 120
Cvic_h          ATKKDPARHSKLEKADILEKTVKHLQELQRQQAAMQQASDPKVINKFKAGFADCANEVSR 120
                **************************************:***::**********.*****

Dmel_h          FPGIEPAQRRRLLQHLSNCINGVKTELHQQQRQQ-----QQQSIHAQMLPSPPSSPEQDS 175
Cvic_h          FPGLEPAVKRRLLQHLQNCINGVKTELHHHQRQASAASVAVQQLHGQILPSPPSSPEQDH 180
                ***:*** :*******.***********::***        *.:*.*:***********

Dmel_h          QQ-GAAAPYLFG--IQQTASGYFLPNGMQVIPTKLPNGSIALVLPQSLPQQQQQQLLQHQ 232
Cvic_h          HHVQQQHQYIMSSQIQQTPHGYFLPNGLQVIPTKLPNGTIALVLPQ---QQQQQQPPQQQ 237
                ::      *::.  ****. *******:**********:*******   ******  *:*

Dmel_h          QQQQQLAVAAAAAAAAAAQQQPMLVSMPQRTASTGSASSHSSAGYESAPGSSSSCSYAPP 292
Cvic_h          -------------------AAPMLVPIPSRTASTGSASSHSSY-EQSYSSNSAMMHFAPP 277
                                     ****.:*.*************   :* ...*:   :***

Dmel_h          SPANSSYEPMDIKPSVIQRVPMEQ------QPLSLVIKKQIKEEEQPWRPW 337
Cvic_h          SPAN-SHEAMDYKPSVIHHAPAQQHYHNNQQPLALVTKKQIKEEEQPWRPW 327
                **** *:*.** *****::.* :*      ***:** **************
```

b

```
Dmel_emc        MKSLTAVCQTGASGMPALNASGRIQRHPTHRGDGENAEMKMYLSKLKDLVPFMPKNRKLT 60
Cvic_emc        MKSLTAVCQTGAS-MPAINPNGRINRPPHHRGDGENAEMKMYLSKLKDLVPFMPKNRKLS 59
                ************* ***:*..***:* * ******************************:

Dmel_emc        KLEIIQHVIDYICDLQTELETHPEMGNFDAAAALTAVNGLHEDEDSDMEDADAEAEAEVD 120
Cvic_emc        KLEIIQHVIDYICDLQSELETHPEMSNFDAAAALNFTANLERVTAAVNNGHDDEGDDEED 119
                ****************:********.********. . .*..   :  :. * *.: * *

Dmel_emc        PDILA---QRLN-AEQPAKVSSPAARLPLTDRQTPNTLVAPAHPQQHQQQQQLQLQQQQL 176
Cvic_emc        DEMYEGIQERLNGSMQLQQQSSPAQRQPLVDRQTPNTILPATQQQQQQQMIVSAVNNTTA 179
                 ::     :*** : *  : **** * **.*******::..:: **:**     :::

Dmel_emc        QSQQQLSNSLATPQNAEKDSRQS----------------------------- 199
Cvic_emc        TSNSITTNSLAAVTSSSSSTAAATTAATTTTAAATNLSSHTVGFHCKKNMVL 231
                 *:.  :****:  .:...:  :
```
