# Supplementary figures and images for: The bristle patterning genes hairy and extramacrochaetae regulate the development of structures required for flight in Diptera
Source: Dev Biol. 2014 Apr 15;388(2):205–15. doi: 10.1016/j.ydbio.2013.12.032 (PMC3988846; doi:10.1016/j.ydbio.2013.12.032)

SUPP. FIGURE 2

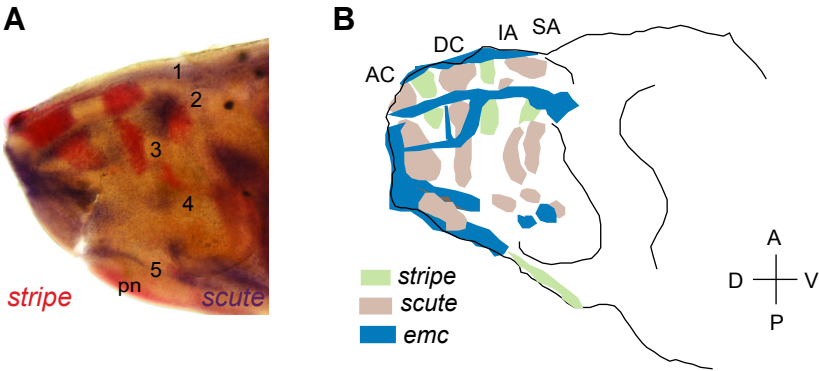

Supplement: Supplementary file 2 — Supplementary Material Supplementary Figure 2 Expression of extramacrochaetae and stripeB in Calliphora vicina, visualized by double in situ hybridization. (A) Double in situ hybridization showing the expression domains for extramacrochaetae (blue) and stripe (red) in the presumptive thorax at 2 h APF. (B) Drawing of the thoracic disc indicating the expression domains of scute (brown), extramacrochaetae (blue) and of stripe (green). extramacrochaetae and stripe are mostly complementary. AC, acrostichal; DC, dorsocentral; IA, intraalar; SA, supraalar; pn, postnotum [file mmc2.pdf]
